# Antibacterial Peptides

**DOI:** 10.3390/antibiotics9040142

**Published:** 2020-03-26

**Authors:** Jean-Marc Sabatier

**Affiliations:** Université Aix-Marseille, Institut de Neurophysiopathologie (INP), UMR 7051, 13005 Marseille, France; sabatier.jm1@libertysurf.fr

As natural host defense compounds produced by numerous prokaryotic and eukaryotic life forms, antimicrobial peptides (AMPs) are now emerging as solid candidate chemotherapeutic drugs to fight against the various types of pathogenic Gram-positive and Gram-negative bacteria, especially those resistant to current antibiotics. This special issue of *‘Antibiotics’* has been focused on the various aspects of such AMPs, from their discovery to the structural and functional characterization thereof. The authors of articles published in this special issue (10 articles, including a review article) are thanked for their important contributions to this essential field of applied research, by allowing a more ‘in-depth’ knowledge on the AMPs.

A first original article by Lattorff deals with the social environment-dependency of two lysozyme genes expression in bumblebees (lysozyme being part of the antimicrobial response of these insects), as well as its tissue specificity [1]. Boix-Lemonche and collaborators [2] developed an interesting fast fluorescence-based microplate assay to examine the effects of AMPs on membranes of whole Gram-positive bacteria. Apart from providing a tool to investigate the mode of action of antibacterials on Gram-positive bacteria, this approach might be particularly useful to screen novel AMPs. Other key studies by Flórez-Castillo [3], Della Pelle [4], Paquette [5], and their collaborators have reported on the structural properties, molecular docking simulation experiments, and/or antibacterial potential of specific antimicrobials, i.e., Ib-M6, Antarctic fish (transcriptome-derived) Trematocine, and *E. coli* antimicrobial molecule, respectively. The data presented are of great interest in the field and may help the design of potent candidate AMPs. Shelenkov and coworkers [6], by performing a computer-based search for potential AMPs in 1267 plant transcriptomes (50–150 peptides were highlighted in each transcriptome), also provided us with a large number of candidate AMPs to examine. By peptide/protein engineering, some ‘optimized’ chemical structures of AMPs can be selected and chemically produced, an approach used by Liscano et al. with Alyteserin 1c [7] and Woodburn et al. [8]. Such antibacterial compounds were shown to possess distinct potencies and/or selectivities toward Gram-positive and Gram-negative bacteria and may lead to newly designed AMP(s) with potent activity on antibiotic-resistant bacterial strain(s) [8]. Importantly, Cheng and collaborators [9] found that a scorpion venom defensin (BmKDfsin3, a host defense antimicrobial peptide) was able to dose-dependently inhibit Hepatitis C viral infection of target cells via suppression of the p38 mitogen-activated protein kinase (MAPK) activation. Finally, an outstanding up-to-date review article by Gray and Wenzel [10] on the marketed cyclic lipopeptide antibiotic Daptomycin is provided in this special issue on ‘Antimicrobial peptides’. I strongly believe that the scientists and clinicians working in the field will find the special issue of particular interest and a real source of inspiration.
